# Light Emitting Diodes Photobiomodulation Improves Cardiac Function by Promoting ATP Synthesis in Mice With Heart Failure

**DOI:** 10.3389/fcvm.2021.753664

**Published:** 2021-12-02

**Authors:** Wenwen Zhang, Xinlu Gao, Xiuxiu Wang, Desheng Li, Yiming Zhao, Tingting Zhang, Jingwen Ne, Binbin Xu, Shuainan Li, Zuke Jiang, Hongyue Sun, Wenya Ma, Fan Yang, Benzhi Cai, Baofeng Yang

**Affiliations:** ^1^Department of Pharmacology (State-Province Key Laboratories of Biomedicine-Pharmaceutics of China, Key Laboratory of Cardiovascular Research, Ministry of Education), College of Pharmacy, Harbin Medical University, Harbin, China; ^2^Northern Translational Medicine Research and Cooperation Center, Heilongjiang Academy of Medical Sciences, Harbin Medical University, Harbin, China; ^3^Institute of Clinical Pharmacy, The Heilongjiang Key Laboratory of Drug Research, Harbin Medical University, Harbin, China

**Keywords:** light emitting diodes (LED), light emitting diodes-based therapy (LEDT), heart failure (HF), cardiomyocyte contractility, adenosine triphosphate (ATP)

## Abstract

Heart failure (HF) is the common consequences of various cardiovascular diseases, often leading to severe cardiac output deficits with a high morbidity and mortality. In recent years, light emitting diodes-based therapy (LEDT) has been widely used in multiple cardiac diseases, while its modulatory effects on cardiac function with HF still remain unclear. Therefore, the objective of this study was to investigate the effects of LED-Red irradiation on cardiac function in mice with HF and to reveal its mechanisms. In this study, we constructed a mouse model of HF. We found that LED-Red (630 nm) was an effective wavelength for the treatment of HF. Meanwhile, the application of LED-Red therapy to treat HF mice improved cardiac function, ameliorate heart morphology, reduced pulmonary edema, as well as inhibited collagen deposition. Moreover, LED-Red therapy attenuated the extent of perivascular fibrosis. Besides, LED-Red irradiation promoted calcium transients in cardiomyocytes as well as upregulated ATP synthesis, which may have positive implications for contractile function in mice with HF. Collectively, we identified that LED-Red exerts beneficial effects on cardiac function in HF mice possibly by promoting the synthesis of ATP.

## Introduction

Heart failure (HF), the common consequences of various cardiovascular diseases, has a high mortality and morbidity rate and occurs mainly in middle-aged and elderly people, and it characterized clinically by low cardiac output and edema (1, 2). The mechanisms for HF are complicated and are related to cardiac pathological remodeling and electrophysiological disturbances (3). A series of pathological changes, such as myocardial fibrosis, excitation-contraction coupling, and metabolic dysfunction, are involved in the development and progression of HF (4). A recent study presented that the increased levels of sarcoplasmic reticulumcalciumATPase 2a (SERCA2a) protein led to enhanced cardiac contractility in HF mice (5). Increasing the rate of fatty acid oxidation and reducing glycolysis in cardiac myocytes can promote ATP production and contributes to delaying the deterioration of cardiac function with HF (6, 7). For the pathogenesis of HF, the current treatment is mainly pharmacological and interventional (7, 8). However, some HF therapeutic drugs have been shown to cause undesirable side effects, which in turn can lead to an increased burden on the body (9). Therefore, new therapeutic approaches are necessary for patients with HF.

Photobiomodulation is a new technology and has been used for the treatment of many diseases such as tumors, degenerative diseases, muscle injuries in recent years (10–12). Light emitting diodes (LED), as incoherent emitters, are gradually replacing lasers and other light sources due to their wide range of available wavelengths, low heat production, and high photovoltaic conversion rates, making them available for the usage in health and medical applications. Notably, light-emitting diodes-based therapy (LEDT) is increasingly used in cardiovascular field because of its non-invasive, low-cost, and easy-to-use. In recent years, pulsed lasers have been shown to stimulate neurons and cardiomyocytes to generate action potentials (10). Jenkins et al. showed that optical pacing of the heart was performed and accomplished *in vivo* (13). Moreover, the incidence of cardiac arrhythmias in the injured heart was profoundly reduced after treatment with LED irradiation (14). In addition, studies have shown that LEDT could reduce muscle damage, modulate the fiber balance in gastrocnemius muscle tissue, and promote myotubular regeneration (15, 16). Capalonga et al. demonstrated that LED-Red could significantly reduce fatigue and exercise intolerance (17). Considering the cardiovascular effects of photobiomodulation, LEDT might be an effective therapy for HF. Therefore, in the present study, we first observed the effect of LED light source on cardiac function in HF mice. Meanwhile, we conducted a preliminary exploration of the molecular mechanism by which LED exerted their regulatory effects, and designed a promising application scheme for LEDT.

## Materials and Methods

### Animals

C57BL/6 mice (8–10 weeks old) were obtained from the Animal Center of the Second Affiliated Hospital of Harbin Medical University. The use of animals was approved by the Ethic Committee of Harbin Medical University, and all research protocols were in accordance with the Guide for the Care and Use of Laboratory Animals published by the National Institutes of Health. HF models in adult mice were generated by left anterior descending artery (LAD) ligation and transaortic constriction (TAC).

### Establishment of Mouse Models of HF by Left Anterior Descending Artery Ligation

The mice (8–10 weeks old) were anesthetized with 2% avertin (0.1 mL/10 g, i.p.). The animals were orally intubated with a 20—gauge tube and ventilated with a respiratory rate of 100 breaths/min and a tidal volume of 0.3 mL (Mouse Ventilator, UGO BASILE, Biological Research Apparatus, Italy). The LAD was ligated with a 7- 0 prolene sutures. Four weeks after surgery, echocardiography was used to determine whether a mouse model of HF was successfully established. The HF mice were randomly grouped into HF and the HF+LED-Red groups. The operators were all blinded to the experimental grouping as well as other assessments of the experiments.

### Construction of Mouse Models of HF by Transaortic Constriction

Another mouse model of HF was constructed by transaortic constriction (TAC) for the isolation of cardiomyocytes. Adult mice were anesthetized with 2% avertin (0.1 mL/10 g, i.p.). In the TAC model, the transverse aorta of the mice was ligated with a 7–0 prolene sutures so that it was tightly attached to a 27—gauge needle between the carotid arteries. Then, the needle was promptly removed to yield a 0.4 mm diameter constriction. For the sham group of mice, the animals received the same procedures but without aorta constriction. The HF mice were randomly grouped into the HF and the HF+ LED-Red groups.

### LED Phototherapy Protocol

In animal experiments, four weeks after HF surgery, a 7—day phototherapy regimen was started in the HF + LED-Red group. The operation was performed as follows. The LED-Red group mice were manually immobilized on a manipulator table. LED-Red was applied dermally fifth ribs of the left thorax of the mice to create a prototype light spot of 1 cm^2^. The mice in the HF + LED-Red group were subjected to LED-Red (630 nm; 3 mW/cm^2^) irradiation 7 times/week for 10 min each time. Subsequently, a second echocardiographic analysis was applied to assess the cardiac function of the mice. In vitor irradiated experiments, cardiomyocytes from neonatal mouse and adult HF mice (TAC model) were irradiated with LED-Red (630 nm, 2.5 mW/cm^2^), for 10 min or placed in the dark, and various analyses were performed immediately after irradiation.

### Cardiac Echocardiography

Mice were anesthetized with 2% avertin (0.1 mL/10 g, i.p.). Echocardiography was performed by using a Vevo770 imaging system (VisualSonics, Toronto, Canada) with a 40 MHz probe. Two-dimensional short-axis views were applied to image the hearts of mice under general anesthesia at the level of maximum left ventricular diameter. Left ventricular internal dimension end-diastole (LVID; d), left ventricular internal dimension end-systole (LVID; s), left ventricular ejection fraction (LVEF), and left ventricular fractional shortening (LVFS) were measured by left ventricular M-mode recordings.

### Immunohistochemistry Assay

Hearts were perfused with 4% paraformaldehyde (PFA) and then fixed at room temperature (RT) for 15 min. After rinsing with PBS, the hearts were embedded in optimal cutting temperature compound (OCT). The frozen tissue blocks were then frozen at −80°C for at least 24 h. For the detection of angiogenesis, the ultra-sensitive TMSP kit (KIT-9710, Maixin, China), von Willebrand Factor (vWF) kit (65707, Cell Signaling Technology, USA) and DAB kit (ZLI-9017, Zhongsanjincheng, China) were performed on transversal 10-mm-thick sections. Images were acquired using a confocal laser scanning microscope (FV300; Olympus, Japan).

### Histology

Five weeks after HF surgery, the hearts were collected and perfused with 4% PFA, and then fixed at 4°C for 24 h. Subsequently, the hearts were dehydrated and sealed in paraffin. To quantify the area of left ventricular fibrosis, transversal 4-mm-thick sections were performed with Masson's trichrome (G1340, Solarbio, China). The cardiac morphology was determined on 4-mm-thick vertical sections using hematoxylin and eosin (HE) staining kit (G1120, Solarbio, China). Staining procedures for all sections were performed according to the manufacturer's protocol. Sections were visualized under a microscope (Axio Vert.A1, Zeiss, Germany).

### HUVECs Culture and Tube Formation Assay

Human umbilical vein endothelial cells (HUVECs, 1.5 × 10^4^ cells) were plated on 200 μL matrigel (354230, BD biosciences, USA) in 24-well plates. After the cells were attached, HUVECs were irradiated with LED-Red (630 nm, 2.5 mw/cm^2^) for 10 min or placed in the dark. After incubation at 37°C and 5% CO_2_ for 2, 4, and 6 h, images of tube structures were taken with a phase contrast microscope (TS100, Nikon, Japan).

### Isolation of Cardiomyocytes

Hearts were isolated from mice within 2 days of birth. Then, hearts were cut into pieces, digested with trypsin (C0202, Beyotime, China), centrifuged (1300 rpm, 5 min, 4°C) and cells were collected. Cells were suspended in containing HIGH GLUCOSE medium (D6429, Sigma, USA), 10% Fetal bovine serum, 1% penicillin-streptomycin (V900929, Sigma, USA) for 1.5 h at 5% CO_2_ and 37°C. The suspended cells were then seeded onto 0.1% gelatin-coated 6-well culture dishes and incubated for 48 h at 5% CO_2_ and 37°C in a humidified atmosphere. Hearts were obtained after euthanasia of mice. After connecting the heart to the Langendorff perfusion apparatus, the hearts were perfused with Ca^2+^-free tyrode buffer (in mM) for 5 min at 37°C: NaCl 137, KCl 5.4, KH_2_PO_4_ 0.16, glucose 10, CaCl_2_ 1.8, MgCl_2_·6H_2_O 0.5, and HEPES 5.0 (adjusted to pH 7.35–7.45 with NaOH 100). The hearts were perfused with collagenases B (1 mg/mL, Gibco, USA) enzymatic solution at 37°C and 50 μM Ca^2+^ was added to digest the tissue. Digestion was terminated as soon as the hearts became flabby, transparent, and pale pink in color, and were quickly removed from the Langendorff perfusion apparatus. The completely digested heart was separated into small pieces and a suspension of left ventricular cardiomyocytes was harvested in 50 μM Ca^2+^. The suspensions were lightly centrifugated (500 rpm, 30 s, 4°C) and loaded into culture chambers for further studies.

### Measurement of Shortening Rate of Cardiomyocytes

Isolated cardiomyocytes were placed in a thermostat and stimulated by 1 Hz electric field to achieve a steady state. After 20 s of stimulation, cardiomyocytes showed stable contraction. Video images of cardiomyocyte contraction process at 4 ms were acquired by line scanning using a Flash4.0 LT camera (4 ms/line; C11440-42U; Hamamatsu, Japan). HCImageLive and Image J were used to measure cardiomyocyte length during systole and diastole. Cardiomyocytes shortening = (diastolic length – systolic length) / diastolic length × 100%.

### Calcium Transient Measurement

Isolated cardiomyocytes were incubated with 5 μM Fluo-3 (F1242, Invitrogen, USA) and 0.01% Pluronic® F127 (BASF, Florham Park, USA) in Tyrode's solution at 37°C for 30 min, and then the cardiomyocytes were stimulated to contract stably after 1 Hz of live video recording in a thermostatic bath with a high-speed CCD camera for filming.


$$\begin{array}{l} {\text{Ca}}^{\text{2+}}\text{ transient amplitude = }\\ \frac{\text{peak systolic }{\text{Ca}}^{\text{2+}}\text{- diastolic }{\text{Ca2}}^{\text{2+}}}{\text{diastolic }{\text{Ca}}^{\text{2+}}}\text{ = }\frac{\text{F-F0}}{\text{F0}} \end{array}$$

### ATP Content Determination

For the measurements of ATP in cardiomyocytes, the cells were washed and centrifuged (250 rpm, 8 min, 4°C). For myocardial tissue ATP measurements, 0.01 g of tissue was added to 100 μL of ATP extract and homogenized in an ice-water bath. After homogenization of harvested cardiomyocytes and myocardial tissue, ATP content assay kit (BC0305, Solarbio, China) was performed according to the manufacturer's instructions.

### Statistics

All statistical analyses was performed with GraphPad-Prism (version 7.0, GraphPad Software Inc., USA). Data are expressed as mean ± SEM. Statistical analyses were performed using unpaired Student's *t*-test or One-Way Analysis of Variance (ANOVA) followed by Tukey's *post-hoc* analysis. The decay time constants of calcium transients were calculated by monoexponential fitting. *P* < 0.05 was considered statistically significant.

## Results

### LED-Red Therapy Enhances Cardiac Function in Mice With Heart Failure

**Figure 1:**
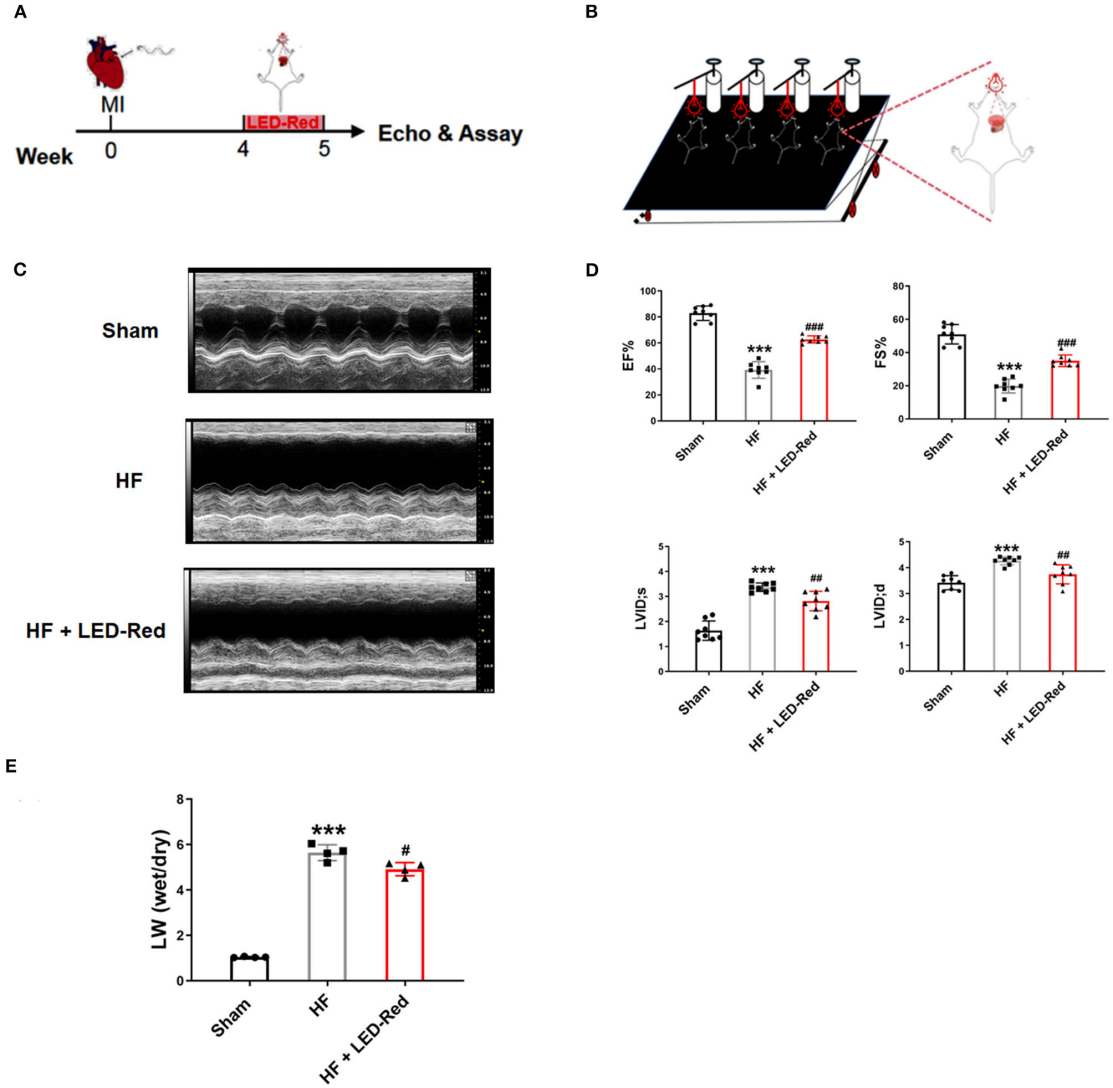
LED-Red therapy improves heart function in mice with heart failure. **(A)** LED-Red therapy protocol. **(B)** Block diagram of the set-up of the LED-Red irradiation experiment. **(C,D)** Cardiac function by echocardiography. **(C)** Representative M-mode echocardiography of the left ventricle. **(D)** EF, Ejection fraction. FS, Fractional shortening; LVID; s, Left ventricular internal dimension end-systole; LVID; d, Left ventricular internal dimension end-diastole; *n* = 8. **(E)** Lung wet weight to dry weight ratio (LW-wet/LW-dry). *n* = 4. ***P < 0.001 vs. Sham, ^#^*P* < 0.05 vs. HF, ^##^*P* < 0.01 vs. HF, ^###^*P* < 0.001 vs. HF. Data are expressed as the mean ± SEM.

To screen for the appropriate wavelength of LED light irradiation to treat HF mice, the HF mice were received either LED-Green (520 nm) or LED-Red (630 nm) irradiation. Echocardiography showed that there was no difference in cardiac function between the HF group and HF + LED-Green group (Supplementary Figure 1A). However, LED-Red irradiation upregulated the EF and FS in the hearts of HF mice (Supplementary Figure 1A). The above results demonstrated that LED-Red could improve myocardial contractile function. It may have a potential regulatory role in inhibiting myocardial injury in HF mice. Therefore, in the following study, we focused on the regulation of cardiac function in HF mice by LED-Red. The effect of LED-Red on cardiac function in HF mice were explored using the designed LED-Red therapy (Figures 1A,B). As expected, the EF and FS were improved in HF mice treated with LED-Red therapy compared to HF mice (Figures 1C,D). Notably, the LVID;s and LVID;d were obviously reduced in the HF + LED-Red group (Figure 1D). These results suggest that LED-Red therapy is sufficient to improve myocardial systolic function in mice with HF. Furthermore, the mice with HF + LED-Red had a milder degree of pulmonary edema compared to the HF group (Figure 1E).

### LED-Red Therapy Inhibits Myocardial Remodeling in Mice With Heart Failure

**Figure 2:**
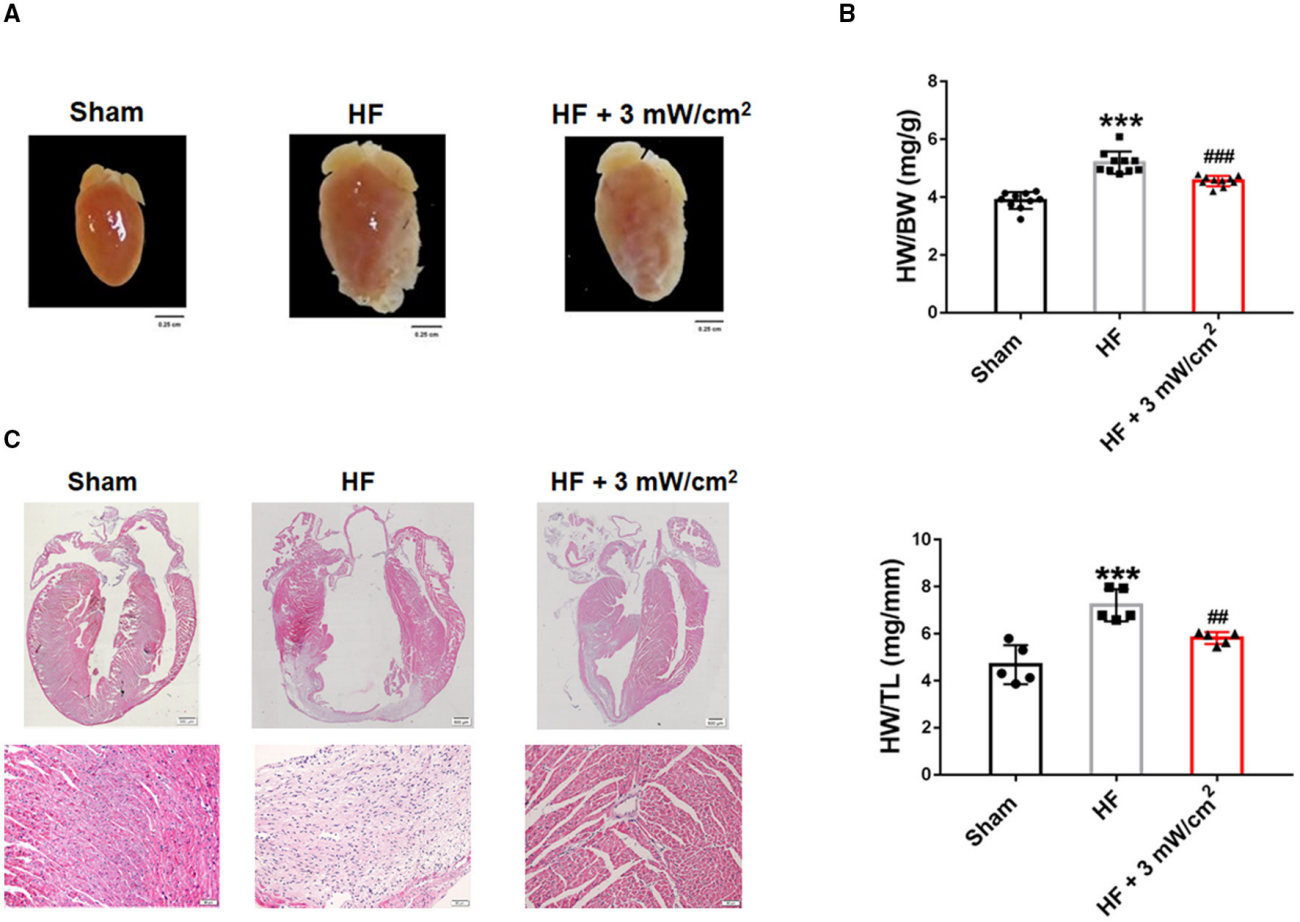
LED-Red therapy inhibits myocardial remodeling in mice with heart failure. **(A)** Gross morphology of hearts. Scale bar = 0.25 cm. **(B)** Heart weight to weight ratio (HW/BW) of mice, *n* = 10. Heart weight to tibia length (HW/TL) of mice, *n* = 5. **(C)** Myocardial remodeling of mice by HE staining. Scale bar = 500 μm (upper panel), 50 μm (lower panel). ***P < 0.001 vs. Sham, ^##^*P* < 0.01 vs. HF. Data are expressed as the mean ± SEM.

To further positive the effects of LED-Red therapy on cardiac structure and morphology in mice with HF, we performed histological and morphological analyses. At 4 weeks after LAD ligation, the hearts of HF mice showed an overall abnormal enlargement along with a thinning of the ventricular wall compared to Sham mice. These cardiac pathological changes were improved after treatment with LED-Red irradiation (Figure 2A). Results that the HW/BW and heart weight to tibia length (HW/TL) of LED-Red treated mice had a downregulated effect relative to HF mice (Figure 2B). Morphologically, hematoxylin-eosin (HE) staining showed that myocardial tissue disorders were reduced in the HF+LED-Red group of mice (Figure 2C). In summary, LED-Red reduced pathological cardiac hypertrophy and disorganization of myocardial tissue structure in HF mice.

### LED-Red Therapy Reduces Fibrosis With Heart Failure

**Figure 3:**
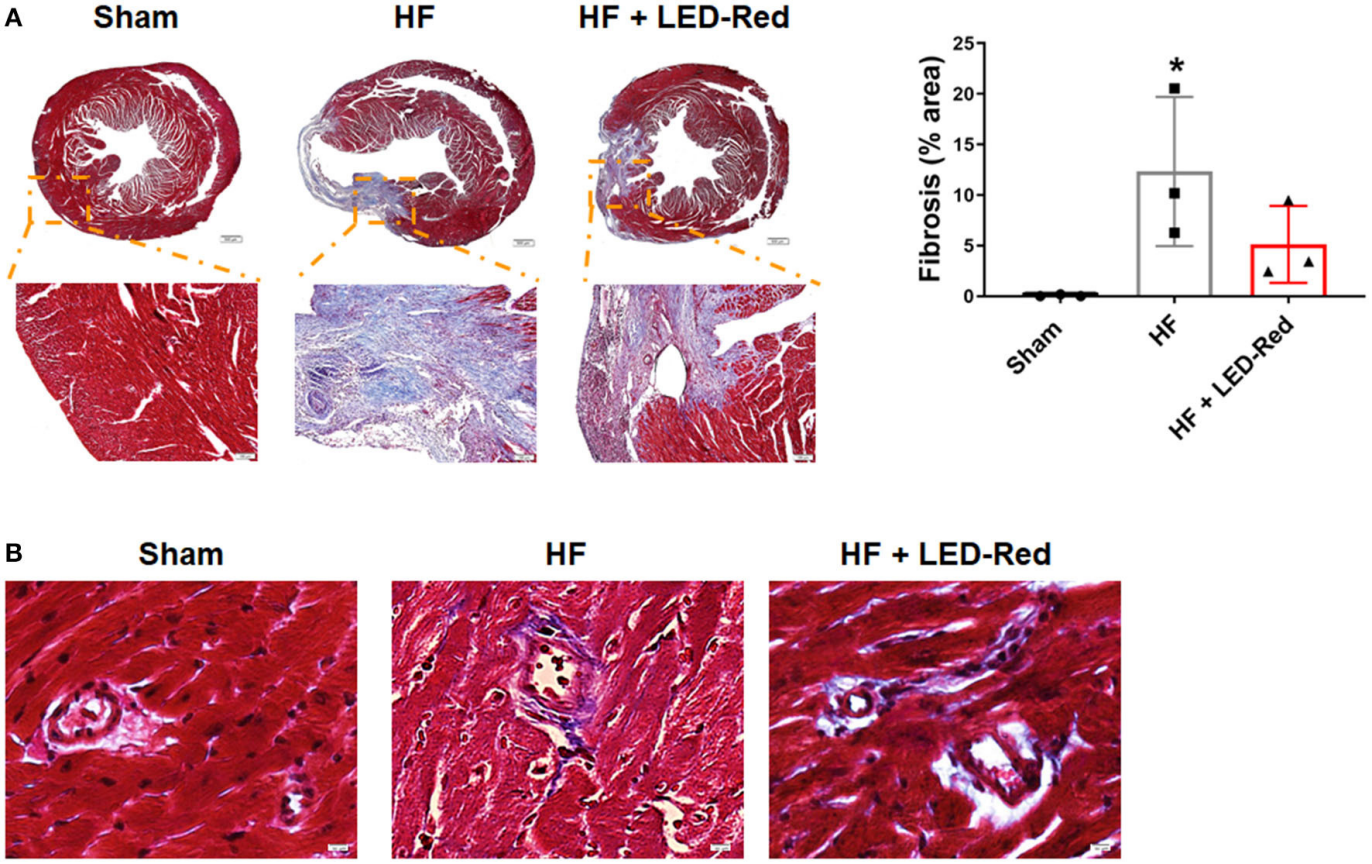
LED-Red therapy reduces fibrosis with heart failure. **(A)** Fibrosis of mice by Masson staining. Scale bar = 500 μm (upper panel), 100 μm (lower panel). **(B)** The perivascular fibrosis by Masson staining. Scale bar = 50 μm. *n* = 3, *P < 0.05 vs. Sham. Data are expressed as the mean ± SEM.

Next, to determine the positive effect of LED-Red therapy on HF, we examined the changes in collagen deposition in the myocardium of HF mice. Masson staining confirmed that LED-Red therapy reduced the volume of collagen in the myocardium (Figure 3A). In addition, we explored whether LED-Red therapy had an effect on the structure of blood vessels, which is essential for adequate blood supply to the heart. The perivascular fibrosis in the myocardium was then examined. Result showed that there was a reduction in perivascular collagen in the HF + LED-Red group (Figure 3B).

### LED-Red Therapy Promotes Myocardial Angiogenesis With Heart Failure

**Figure 4:**
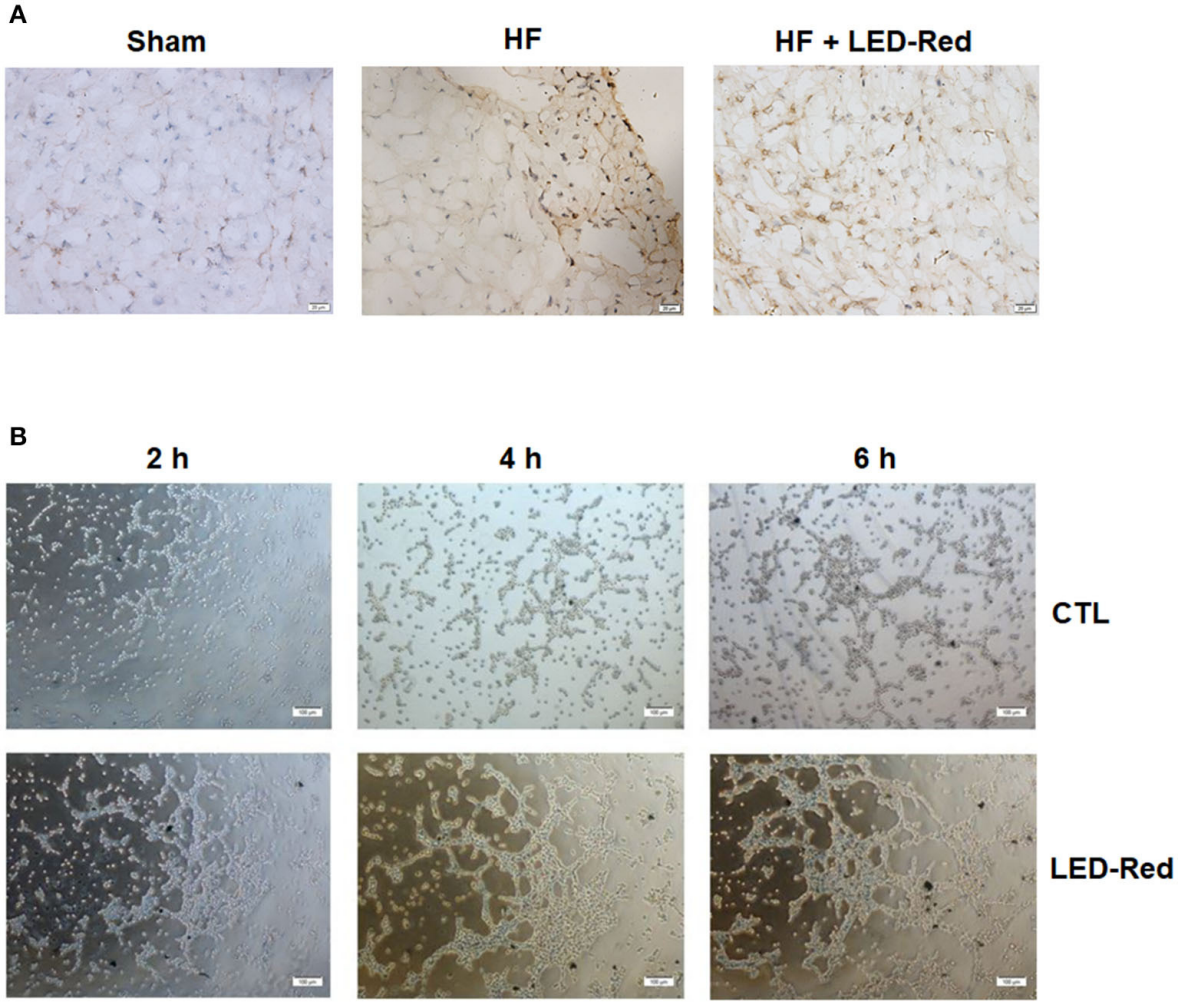
LED-Red therapy promotes angiogenesis with heart failure. **(A)** Angiogenesis of heart in mice after HF by VWF immunohistochemical staining. Scale bar = 20 μm. **(B)** Tube formation in CTL or LED-Red illuminated HUVECs. Scale bar = 100 μm.

To determine whether the LED-Red therapy affects the microvasculature after HF, we performed immunohistochemical staining with vWF. Results showed that the capillary density in the damaged myocardium was increased by LED-Red therapy (Figure 4A). We further demonstrated the effect of LED-Red on angiogenesis *in vitro*, and the results showed that LED-Red therapy enhanced the capillary formation compared with the control group, as demonstrated by the shorter time to tube formation and greater number of capillaries formed in LED-Red-treated HUVECs (Figure 4B). The above results suggest that LED-Red therapy can attenuate unfavorable remodeling and promote angiogenesis.

### LED-Red Irradiation Enhances Cardiomyocyte Contractility

**Figure 5:**
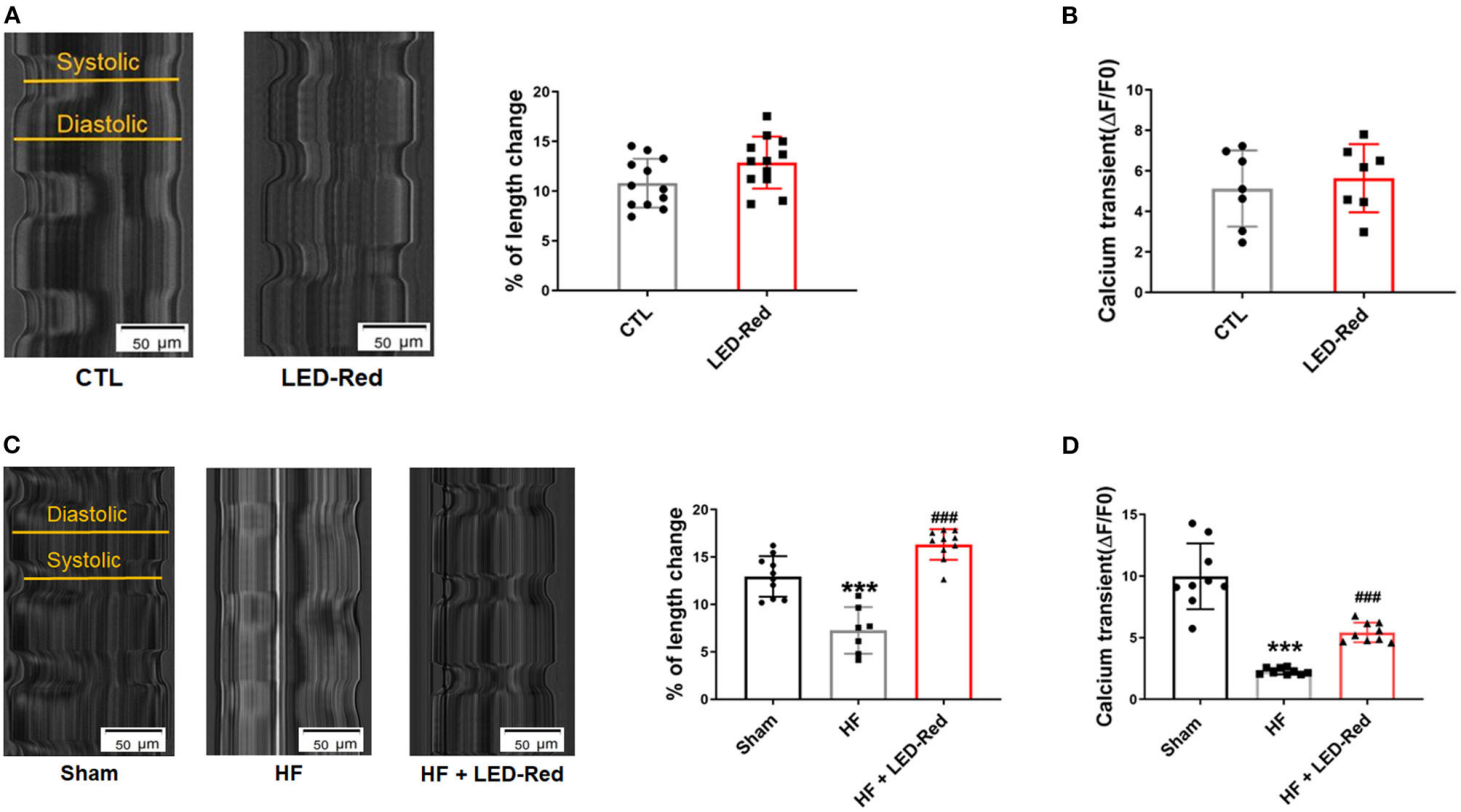
LED-Red irradiation enhances cardiomyocytes contractility. **(A)** Shortening of isolated single ventricular myocytes of mice. Scale bar = 50 μm. *n* = 12. **(B)** Peak calcium transient analysis in isolated single ventricular myocytes of mice, *n* = 7. **(C)** Shortening of isolated single ventricular myocytes of Sham mice or HF mice. Scale bar = 50 μm, *n* = 6. **(D)** Peak calcium transient analysis in isolated single ventricular myocytes of Sham mice or HF mice, *n* = 9. ***P < 0.001 vs. Sham, ^###^*P* < 0.001 vs. HF. Data are expressed as the mean ± SEM.

In the above studies, we found that LED-Red had an enhanced effect on myocardial contractile function in LAD-induced HF mice. Next, we also examined the effects of LED-Red on the contractile function of cultured neonatal mouse cardiomyocytes *in vitro* as well as ventricular cardiomyocytes from TAC-induced HF mice, respectively. The results showed that in neonatal mouse cardiomyocytes, the contraction amplitude of cardiomyocytes triggered by field stimulation was increased under LED-Red irradiation (Figure 5A). However, the calcium transients of LED-Red-treated cardiomyocytes were not different from the control cells (Figure 5B). Therefore, the effect of LED-Red on the contractile function of cardiomyocytes under pathological conditions was further investigated. Under LED-Red therapy, the longitudinal length of isolated single ventricular cardiomyocytes in HF mice increased from 6.9 to 17.1% (Figure 5C). However, the calcium transients were increased in HF + LED-Red ventricular myocytes (Figure 5D). These results indicate that LED-Red therapy may enhances failing cardiomyocyte contractility and improve cardiac function in HF mice.

### LED-Red Irradiation Increases ATP Content in Cardiomyocytes and Cardiac Tissue

**Figure 6:**
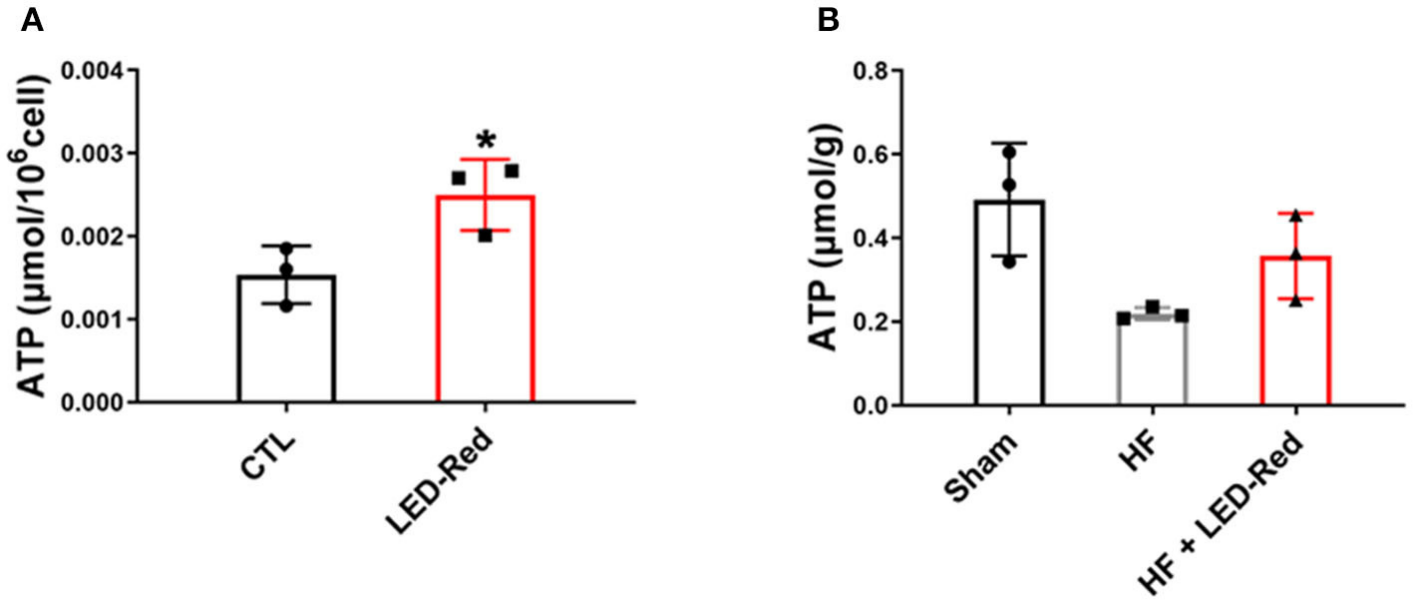
LED-Red irradiation increases ATP content in cardiomyocytes and cardiac tissue. **(A,B)** NADPH assays was applied to measure the content of ATP in isolated ventricular myocytes **(A)**, and cardiac tissue **(B)**. *n* = 3, *P < 0.05 vs. CTL. Data are expressed as the mean ± SEM.

Mitochondria are the main organelle of aerobic respiratory capacity of cardiomyocytes and provide a large amount of ATP to the myocardium during contraction. Therefore, we proformed the NADPH assays to analyze the ATP content in cardiomyocytes and cardiac tissues. Results showed that the ATP levels in LED-Red-treated cardiomyocytes were higher than control cells (Figure 6A). Moreover, the ATP content in cardiac tissues of the HF + LED-Red group was higher relative to the HF group (Figure 6B). These results suggest that LED-Red can induce the ATP synthesis in the mitochondria of cardiomyocytes and cardiac tissue which is important for reducing myocardial injury and delaying the development of HF.

## Discussion

HF is the common consequences of many cardiovascular diseases and is accompanied by severe cardiac output deficit (2). Recently, LEDT has gained significant attention for its therapeutic potential in cardiovascular diseases, especially using low-energy therapy (13, 14). It has been shown that short-term of low-energy visible light does not cause cardiomyocyte damage (18). Furthermore, phototherapy has shown anti-inflammatory effects as well as better functional capacity in HF rats (17). These findings strongly suggest LEDT as a new therapy for HF. In this study, we applied a systematic LED-Red therapy to reverse the decline in cardiac function induced by HF. With the application of this intervention, we demonstrated that the EF and FS were elevated in the HF + LED-Red group compared to those in the HF group. Meanwhile, we found that the ventricular dilation, thinning of the ventricular wall and the area of fibrosis were reduced in the hearts of LED-Red-treated HF mice, suggesting that LED-Red slowed down the myocardial remodeling. These results confirm that the LED-Red therapy is an effective treatment for HF. Previous studies have confirmed that, a range of myocardial lesions are often associated with inadequate blood supply, depending on the structure and function of the blood vessels (3, 19). Therefore, to uncover the function of LED-Red therapy in vascular structure, the perivascular fibrosis and angiogenesis in the myocardium were detected. We observed that LED-Red therapy reduced HF induced perivascular collagen deposition. Consistently, the capillary density in LED-Red therapy-treated hearts and tube formation in LED-Red-treated HUVECs were higher indicating that LED-Red therapy has a positive effect on angiogenesis. It can be speculated that these results are related to the myocardial changes caused by LEDT with modulation of vascular endothelial cell proliferation and collagen deposition (20, 21). Feng et al. found that low-power laser irradiation (632.8 nm) augmented the expression of VEGF, which was the primary pro-angiogenic target for therapeutic angiogenesis (20). This study demonstrates that LEDT can promote the proliferation, migration and tube formation of vascular endothelial cells, which contribute to the ability of phototherapy to enhance angiogenesis (20, 22, 23). Increasing number of basic research and clinical studies have confirmed that phototherapy can also inhibit the accumulation of extracellular matrix and reduce fibrosis (24–26). In conclusion, LED-Red has the effect of slowing down the HF process.

HF is characterized by a reduction in the number of cardiomyocytes along with a decrease in cardiomyocyte contractility. In this study, we reported for the first time that the LED-Red therapy could upregulate calcium handling and cardiac function in single ventricular cardiomyocytes. Cardiomyocytes require large amounts of energy from mitochondria to maintain contractility, and directly use ATP as an energy source, which is the only energy substance. More interestingly, we found that the LED-Red therapy significantly increased ATP levels in cardiomyocytes and cardiac tissue. These data indicate that the LED-Red therapy to increase the synthesis of ATP is important for the enhancement of cardiomyocyte contractile function in the HF heart. However, how cardiomyocytes initiate mitochondria to execute the instruction of ATP production to replenish the damaged myocardium with ATP energy after receiving photonic energy from LED-Red still needs to be further explored. In addition, the mechanism of light-controlled ATP synthesis has been explored in other light therapy research articles. On the one hand, it has been suggested that in the mitochondrial respiratory chain, cytochrome b and c can selectively absorb red light and near infrared light, offering the possibility that phototherapy acts on cardiomyocyte mitochondria to promote ATP synthesis (27, 28). Another study showed that LEDT kept glucose in balance, implying that LED-Red therapy may affect glucose metabolism in HF mice (29, 30). The mechanisms of ATP-induced enhancement of cardiomyocyte contractility are diverse. In terms of calcium handling, increasing ATP enhances the activity of ATPases, including SERCA2a, RyR, and L-type calcium channels, which regulate calcium release. However, considering the complex mechanisms, we speculate that certain LED-Red therapy may modulate the expression or activity of myocardial contractile proteins. Likewise, previous studies have shown that low-energy visible light stimulates an increase in intracellular calcium ion concentrations in cardiomyocytes (18).

It is important to highlight that this study illustrates the positive effect of LED-Red therapy on cardiac function in mice with HF. Furthermore, this finding provides an experimental basis and data support for further research and development of new treatment for HF. However, more studies should be done to try to elucidate the molecular mechanisms underlying this improvement in cardiac function.

## Data Availability Statement

The raw data supporting the conclusions of this article will be made available by the authors, without undue reservation.

## Ethics Statement

The animal study was reviewed and approved by the Ethic Committee of Harbin Medical University.

## Author Contributions

WZ performed experiments, analyzed data, and prepared the manuscript. XG and XW conducted the main experiments and helped analyzed data. DL, YZ, TZ, JN, BX, SL, ZJ, and HS helped perform experiments and collect data. WM helped edit the manuscript. BY, BC, and FY designed the project, oversaw the experiments, and prepared the manuscript. All authors contributed to the article and approved the submitted version.

## Funding

This work was supported by the National Key Research and Development Program of China (2017YFB0403802).

## Conflict of Interest

The authors declare that the research was conducted in the absence of any commercial or financial relationships that could be construed as a potential conflict of interest.

## Publisher's Note

All claims expressed in this article are solely those of the authors and do not necessarily represent those of their affiliated organizations, or those of the publisher, the editors and the reviewers. Any product that may be evaluated in this article, or claim that may be made by its manufacturer, is not guaranteed or endorsed by the publisher.

## Supplementary Material

Supplementary Figure 1 630 nm LED-Red is the appropriate wavelength range for treating heart failure. **(A)** Cardiac function by echocardiography. Ejection fraction, EF. Fractional shortening, FS. *n* = 3, ^***^*P* < 0.001 vs. Sham, ^##^*P* < 0.01 vs. HF. Data are expressed as the mean ± SEM.
