# Supplementary figures and images for: Light Emitting Diodes Photobiomodulation Improves Cardiac Function by Promoting ATP Synthesis in Mice With Heart Failure
Source: Front Cardiovasc Med. 2021 Dec 2;8:753664. doi: 10.3389/fcvm.2021.753664 (PMC8674466; doi:10.3389/fcvm.2021.753664)

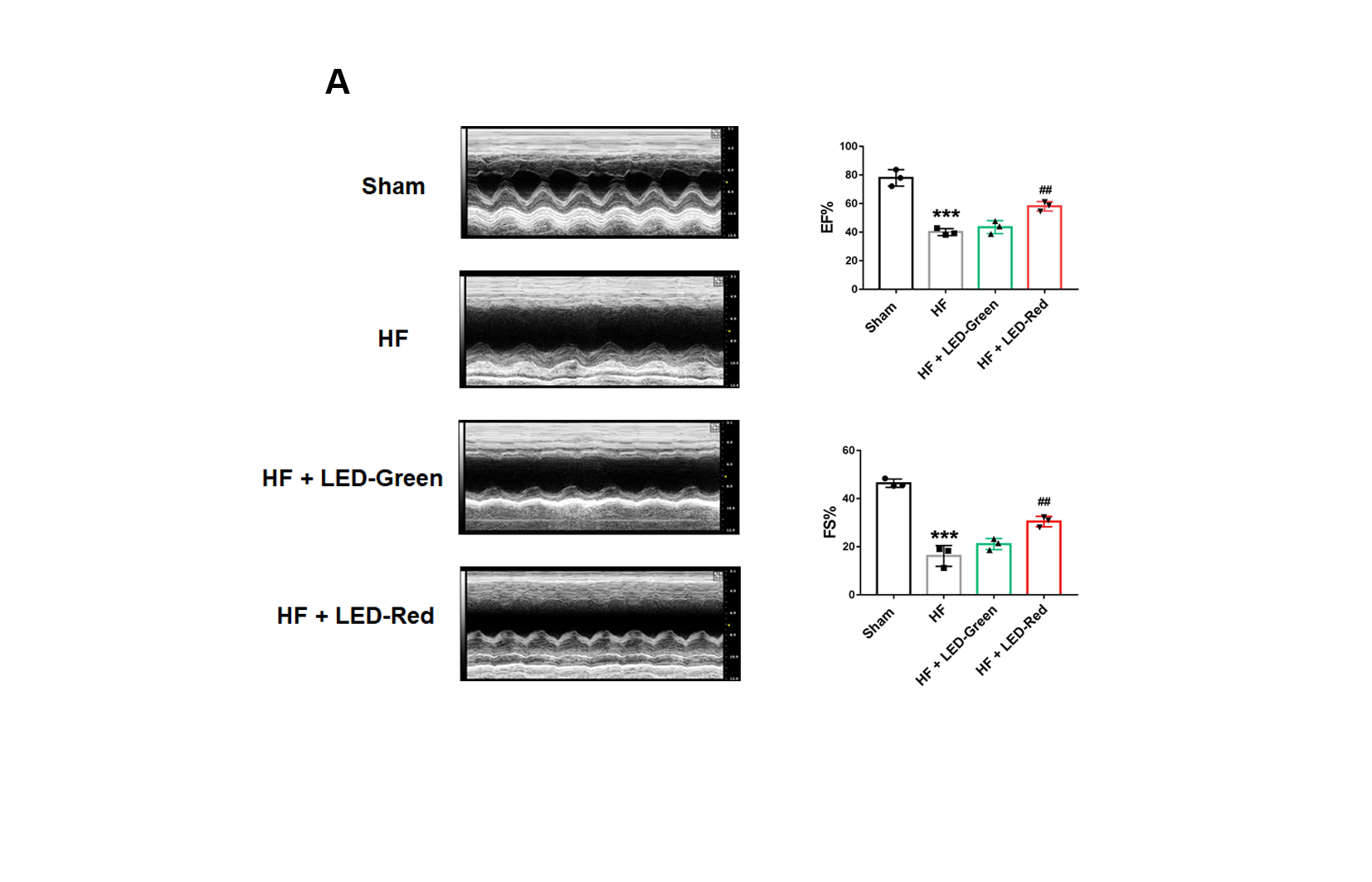

Supplement: Supplementary Figure 1 — 630 nm LED-Red is the appropriate wavelength range for treating heart failure. (A) Cardiac function by echocardiography. Ejection fraction, EF. Fractional shortening, FS. n = 3, ***P < 0.001 vs. Sham, ##P < 0.01 vs. HF. Data are expressed as the mean ± SEM. [file Image_1.tif]
